# Supplementary material for: Noninvasive Mapping of Extracellular Potassium in Breast Tumors via Multi-Wavelength Photoacoustic Imaging
Source: Sensors (Basel). 2025 Jul 31;25(15):4724. doi: 10.3390/s25154724 (PMC12349659; doi:10.3390/s25154724)
Supplement: Supplementary file 1 [file sensors-25-04724-s001.zip › sensors-3733499-supplementary.pdf]

Supporting information

Schematic diagram of SDKNP

The individual components making up the SDKNP are dissolved in 3 mL methanol (0.2 mg SD2, 0.9 mg sodium tetrakis[3,5-bis(trifluoromethyl)phenyl]borate, 1.2 mg valinomycin, 5 mg Pluronic F-127, and 8 mg bis(2-ethylhexyl) sebacate). This methanol cocktail was added dropwise to 30 mL of Millipore water under vigorous stirring. Once added, the surface of the water solution was blasted with argon gas for 1 hour while stirring to remove the methanol. After 1 hour, the self-assembled SDKNPs were concentrated to 20mg/mL using an 100kDa Amicon Ultra-Centrifuge Filter.

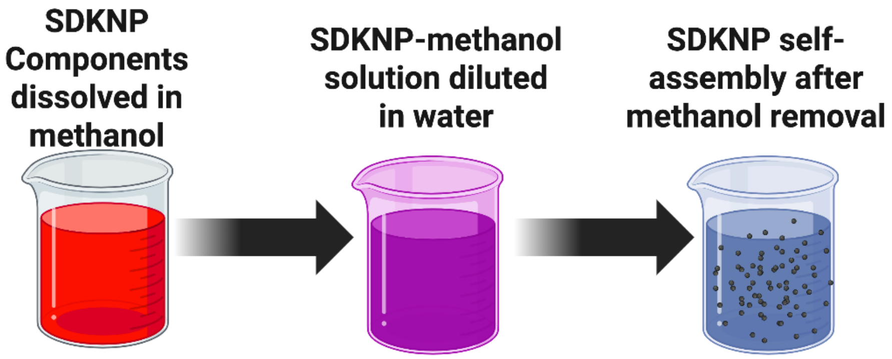

Figure S1. Schematic diagram of SDKNP

Schematic of photoacoustic imaging system

In vivo photoacoustic imaging was conducted using our custom-built dual-modality ultrasound (US) and PAI system, integrated onto a programmable research ultrasound platform (Vantage 256, Verasonics). A linear array transducer (CL15-7, Philips) with a central frequency of 11.25 MHz was employed for both US and PA signal acquisition. For optical excitation, we utilized a tunable optical parametric oscillator (OPO; Slopo, Amplitude) pumped by a Nd:YAG laser (Surelite, Amplitude), delivering 5 ns pulses at a repetition rate of 10 Hz. The system, accelerated by a GPU, enables real-time acquisition of co-registered PA and US images at 10 frames per second—synchronized with the laser pulse rate. At a 6 mm imaging depth, the lateral and axial resolutions were quantified to be approximately 226 μm and 166 μm, respectively. Illumination on the sample surface covered a 2 cm × 3 cm rectangular area, with the optical

fluence maintained around 15 mJ/cm^2 to comply with the ANSI safety limits for human exposure. The system schematic is shown in Supplementary Figure S2.

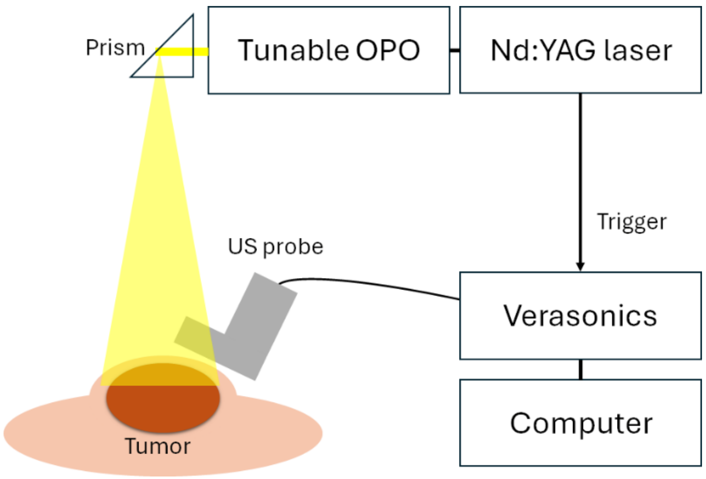

Figure S2. Schematic of photoacoustic imaging system with a tunable OPO laser and Verasonics ultrasound platform.

Method of multi-wavelength PA radiometric imaging of potassium

The ratiometric method using dual wavelengths is significantly affected by endogenous chromophores, such as blood, which produce strong signals at those wavelengths, making accurate measurements difficult. To overcome this limitation, we employed a multi-wavelength PA unmixing approach to determine potassium levels.

$$P_\lambda = k\left[\varepsilon_{HbO_2\_\lambda}\,C_{HbO_2} + \varepsilon_{Hb\_\lambda}\,C_{Hb} + \varepsilon_{NP\_\lambda}\,C_{NP}\right], \qquad (S1)$$

where k is a constant relevant to the *Grüneisen parameter* of the tissue and the sensitivity of the imaging system, ε indicates the absorption coefficient of HbO_2, Hb, or SDKNP at wavelength λ, and C is the concentration of HbO_2, Hb, or SDKNP. As the ratiometric method, the optical absorption of SDKNP at 576 nm, 584 nm, 605 nm or 625 nm can be expressed as a linear function of the optical absorption at the isosbestic point of 560 nm and the K^+ value:

$$\varepsilon_{NP\_\lambda} = \varepsilon_{NP_{\lambda 560}}(\alpha \cdot K^+ + b) \qquad (S2)$$

where the constants α and b for each wavelength (576 nm, 584 nm, 605 nm and 625 nm) can be obtained from ratios of optical absorption as Figure 2. By substituting the NP part in Equation (1) with Equation (2) , the PA signal intensity at all the five wavelengths can be written as:

$$
\begin{aligned}
P_{\lambda560} &= k\left[\varepsilon_{HbO_2\_\lambda560}\, C_{HbO_2} + \varepsilon_{Hb\_\lambda560}\, C_{Hb} + 0\cdot K^{+}\,\varepsilon_{NP\_\lambda560}\, C_{NP} + 1\cdot\varepsilon_{NP\_\lambda560} C_{NP}\right]\\
P_{\lambda576} &= k\left[\varepsilon_{HbO_2\_\lambda576}\, C_{HbO_2} + \varepsilon_{Hb\_\lambda576}\, C_{Hb} + \alpha_{\frac{I_{\lambda576}}{I_{\lambda560}}} K^{+}\,\varepsilon_{NP\_\lambda560}\, C_{NP} + b_{\frac{I_{\lambda576}}{I_{\lambda560}}}\,\varepsilon_{NP\_\lambda560} C_{NP}\right]\\
P_{\lambda584} &= k\left[\varepsilon_{HbO_2\_\lambda584}\, C_{HbO_2} + \varepsilon_{Hb\_\lambda584}\, C_{Hb} + \alpha_{\frac{I_{\lambda584}}{I_{\lambda560}}} K^{+}\,\varepsilon_{NP\_\lambda560}\, C_{NP} + b_{\frac{I_{\lambda584}}{I_{\lambda560}}}\,\varepsilon_{NP\_\lambda560} C_{NP}\right] \qquad \text{(S3)}\\
P_{\lambda605} &= k\left[\varepsilon_{HbO_2\_\lambda605}\, C_{HbO_2} + \varepsilon_{Hb\_\lambda605}\, C_{Hb} + \alpha_{\frac{I_{\lambda605}}{I_{\lambda560}}} K^{+}\,\varepsilon_{NP\_\lambda560}\, C_{NP} + b_{\frac{I_{\lambda605}}{I_{\lambda560}}}\,\varepsilon_{NP\_\lambda560} C_{NP}\right]\\
P_{\lambda625} &= k\left[\varepsilon_{HbO_2\_\lambda625}\, C_{HbO_2} + \varepsilon_{Hb\_\lambda625}\, C_{Hb} + \alpha_{\frac{I_{\lambda625}}{I_{\lambda560}}} K^{+}\,\varepsilon_{NP\_\lambda560}\, C_{NP} + b_{\frac{I_{\lambda625}}{I_{\lambda560}}}\,\varepsilon_{NP\_\lambda560} C_{NP}\right].
\end{aligned}
$$

The Equation (3) can be converted to matrix form:

$$
k\cdot\begin{bmatrix}
\varepsilon_{HbO_2\_\lambda560} & \varepsilon_{Hb\_\lambda560} & 0 & \varepsilon_{NP\_\lambda560}\\
\varepsilon_{HbO_2\_\lambda576} & \varepsilon_{Hb\_\lambda576} & \alpha_{\frac{I_{\lambda576}}{I_{\lambda560}}}\varepsilon_{NP\_\lambda560} & b_{\frac{I_{\lambda576}}{I_{\lambda560}}}\varepsilon_{NP\_\lambda560}\\
\varepsilon_{HbO_2\_\lambda584} & \varepsilon_{Hb\_\lambda584} & \alpha_{\frac{I_{\lambda584}}{I_{\lambda560}}}\varepsilon_{NP\_\lambda560} & b_{\frac{I_{\lambda584}}{I_{\lambda560}}}\varepsilon_{NP\_\lambda560}\\
\varepsilon_{HbO_2\_\lambda605} & \varepsilon_{Hb\_\lambda605} & \alpha_{\frac{I_{\lambda605}}{I_{\lambda560}}}\varepsilon_{NP\_\lambda560} & b_{\frac{I_{\lambda605}}{I_{\lambda560}}}\varepsilon_{NP\_\lambda560}\\
\varepsilon_{HbO_2\_\lambda625} & \varepsilon_{Hb\_\lambda625} & \alpha_{\frac{I_{\lambda625}}{I_{\lambda560}}}\varepsilon_{NP\_\lambda560} & b_{\frac{I_{\lambda625}}{I_{\lambda560}}}\varepsilon_{NP\_\lambda560}
\end{bmatrix}
\begin{bmatrix} C_{HbO_2}\\ C_{Hb}\\ K^{+}\cdot C_{NP}\\ C_{NP}\end{bmatrix}
=
\begin{bmatrix} P_{\lambda560}\\ P_{\lambda576}\\ P_{\lambda584}\\ P_{\lambda605}\\ P_{\lambda625}\end{bmatrix}. \qquad \text{(S4)}
$$

The matrix Equation (4) can be solved by applying the inverse of the matrix.

$$
k\cdot\begin{bmatrix} C_{HbO_2}\\ C_{Hb}\\ K^{+}\cdot C_{NP}\cdot\varepsilon_{NP\_\lambda560}\\ C_{NP}\cdot\varepsilon_{NP\_\lambda560}\end{bmatrix}
=
\begin{bmatrix}
\varepsilon_{HbO_2\_\lambda560} & \varepsilon_{Hb\_\lambda560} & 0 & 1\\
\varepsilon_{HbO_2\_\lambda576} & \varepsilon_{Hb\_\lambda576} & \alpha_{\frac{I_{\lambda576}}{I_{\lambda560}}} & b_{\frac{I_{\lambda576}}{I_{\lambda560}}}\\
\varepsilon_{HbO_2\_\lambda584} & \varepsilon_{Hb\_\lambda584} & \alpha_{\frac{I_{\lambda584}}{I_{\lambda560}}} & b_{\frac{I_{\lambda584}}{I_{\lambda560}}}\\
\varepsilon_{HbO_2\_\lambda605} & \varepsilon_{Hb\_\lambda605} & \alpha_{\frac{I_{\lambda605}}{I_{\lambda560}}} & b_{\frac{I_{\lambda605}}{I_{\lambda560}}}\\
\varepsilon_{HbO_2\_\lambda625} & \varepsilon_{Hb\_\lambda625} & \alpha_{\frac{I_{\lambda625}}{I_{\lambda560}}} & b_{\frac{I_{\lambda625}}{I_{\lambda560}}}
\end{bmatrix}^{-1}
\begin{bmatrix} P_{\lambda560}\\ P_{\lambda576}\\ P_{\lambda584}\\ P_{\lambda605}\\ P_{\lambda625}\end{bmatrix}. \qquad \text{(S5)}
$$

From the Equation (5), the 4×1 matrix of left side of Equation (5) can be computed when all the variables on the right side of the equation are known or can be determined by multi-wavelength PA measurements. Then K^+ level of each pixel in the imaging plane can be calculated by

$$
K^{+} = \frac{k\cdot K^{+}\cdot C_{NP}\cdot\varepsilon_{NP\_\lambda560}}{k\cdot C_{NP}\cdot\varepsilon_{NP\_\lambda560}}\ , \qquad \text{(S6)}
$$

where both k and $\varepsilon_{NP_{\lambda560}}$ are constants. MATLAB (R2022b, Mathworks, Natick, MA) was used for calculating K^+ imaging results from multiple PA images.
